# A comprehensive in silico exploration of the impacts of missense variants on two different conformations of human pirin protein

**DOI:** 10.1186/s42269-022-00917-7

**Published:** 2022-07-30

**Authors:** Auroni Semonti Khan, Nahid Parvez, Tamim Ahsan, Sabrina Samad Shoily, Abu Ashfaqur Sajib

**Affiliations:** 1 Department of Genetic Engineering and Biotechnology, Jagannath University, Dhaka, 1100 Bangladesh; 2 Molecular Biotechnology Division, National Institute of Biotechnology, Savar, Dhaka, 1349 Bangladesh; 3 Department of Genetic Engineering and Biotechnology, University of Dhaka, Dhaka, 1000 Bangladesh

**Keywords:** Pirin, Pathogenic variants, Non-heme protein, Inflammation, Transcriptional regulation, Oxidative stress, Cancer, NFκB pathway

## Abstract

**Background:**

Pirin, a member of the cupin superfamily, is an iron-binding non-heme protein. It acts as a coregulator of several transcription factors, especially the members of NFκB transcription factor family. Based on the redox state of its iron cofactor, it can assume two different conformations and thereby act as a redox sensor inside the nucleus. Previous studies suggested that pirin may be associated with cancer, inflammatory diseases as well as COVID-19 severities. Hence, it is important to explore the pathogenicity of its missense variants. In this study, we used a number of in silico tools to investigate the effects of missense variants of pirin on its structure, stability, metal cofactor binding affinity and interactions with partner proteins. In addition, we used protein dynamics simulation to elucidate the effects of selected variants on its dynamics. Furthermore, we calculated the frequencies of haplotypes containing pirin missense variants across five major super-populations (African, Admixed American, East Asian, European and South Asian).

**Results:**

Among a total of 153 missense variants of pirin, 45 were uniformly predicted to be pathogenic. Of these, seven variants can be considered for further experimental studies. Variants R59P and L116P were predicted to significantly destabilize and damage pirin structure, substantially reduce its affinity to its binding partners and alter pirin residue fluctuation profile via changing the flexibility of several key residues. Additionally, variants R59Q, F78V, G98D, V151D and L220P were found to impact pirin structure and function in multiple ways. As no haplotype was identified to be harboring more than one missense variant, further interrogation of the individual effects of these seven missense variants is highly recommended.

**Conclusions:**

Pirin is involved in the transcriptional regulation of several genes and can play an important role in inflammatory responses. The variants predicted to be pathogenic in this study may thus contribute to a better understanding of the underlying molecular mechanisms of various inflammatory diseases. Future studies should be focused on clarifying if any of these variants can be used as disease biomarkers.

**Supplementary Information:**

The online version contains supplementary material available at 10.1186/s42269-022-00917-7.

## Background

Pirin (PIR) is a highly conserved protein across both eukaryotes and prokaryotes (Dunwell et al. 2001). It is widely present in sub-nuclear structures of cells (Pang et al. 2004). This protein is predominantly expressed in human liver, heart, kidneys, and muscles while low levels of expression are observed in brain and lungs (Wendler et al. 1997; Pang et al. 2004). Human pirin has been implicated in skin, breast, lung, head and neck, gastrointestinal, cervical cancers (Perez-Dominguez et al. 2021) and tumors of epithelial tissues, hematopoietic and neurological systems (Yoshikawa et al. 2004; Licciulli et al. 2010a; Jungk et al. 2016).

The human pirin is a non-heme iron-binding protein that comprises 290 amino acids and has a molecular weight of 32 kDa. Due to its primary sequence and structural similarity, pirin belongs to the cupin superfamily. It contains two structurally similar β-barrel domains facing each other (Wendler et al. 1997; Pang et al. 2004). The N-terminal domain is highly conserved in mammals, plants, fungi and prokaryotic organisms (Wendler et al. 1997), whereas the C-terminal domain varies. The C-terminal domain does not contain any metal-binding site, but has a cavity which is more compact than that of the N-terminal domain (Pang et al. 2004). The iron cofactor (Fe^2+^ or Fe^3+^) is coordinated by residues His-56, His-58, His-101 and Glu-103 within the negatively charged metal-binding cavity of the N-terminal domain (Pang et al. 2004; Liu et al. 2013). Depending on the redox state, this iron center within the R-shaped surface area allosterically controls the interactions between pirin and its binding partners (Pang et al. 2004; Liu et al. 2013).

Pirin was originally identified as an interactor of nuclear factor I/CCAAT box transcription factor (NFI/CTF1), which can regulate transcription initiation and DNA replication (Santoro et al. 1988; Wendler et al. 1997). Nuclear factor κB (NF-κB), a family of transcription factors, plays crucial roles in intracellular signaling for immune responses and consists of dimeric RelA (p65), RelB, c-Rel, p50, and p52 (Ghosh et al. 1998; Li and Verma 2002). Pirin functions as a transcriptional coregulator for the expression of NF-κB target genes through regulating the binding of NF-κB p65 to κB-sites (Liu et al. 2013). Nuclear translocation of NF-κB and its binding to DNA is prevented by members of the inhibitor of kappa-B (IκB) family (Bhatt and Ghosh 2014). B-cell lymphoma 3-encoded protein (BCL3), despite being a member of the IκB family, has both transactivation and transrepression roles in regulation of p50 or p52 (members of NF-κB family) homodimers mediated pathways (Lenardo and Siebenlist 1994; Maldonado and Melendez-Zajgla 2011). The difference in mechanism of inhibition by IκB and stimulation by BCL3 is not known, but it is evident that these bind to different protein partners. Pirin is one of the four binding partners of BCL3 and is known to enhance the DNA binding by BCL3-p50 via formation of a quaternary complex (Dechend et al. 1999).

The nuclear factor erythroid-derived 2-like 2 (Nrf2) transcription factor triggered in response to cellular oxidative stress conditions can modulate *PIR* gene expression via the functional antioxidant response elements (AREs) in its promoter region (Hübner et al. 2009; Chorley et al. 2012). Chronic cigarette smoking causes increased oxidative stress, which up-regulates expression of pirin and it has been implicated in bronchial epithelial cell apoptosis (Hübner et al. 2009; Chorley et al. 2012). Pirin is an oxidative stress sensor (Perez-Dominguez et al. 2021) and ferric conformation of this protein facilitates binding of NF-κB p65 to DNA (Liu et al. 2013). Although elevated activation of p65 is observed in chronic inflammation (Giridharan and Srinivasan 2018), canonical NF-κB pathway mediated by p65/p50 heterodimer is critically important for innate immunity and inflammatory responses (Oeckinghaus and Ghosh 2009; Liu et al. 2017). Furthermore, a recent study suggested possible association of pirin with the severity of COVID-19 (Shoily et al. 2021).

Pirin may be functionally important and associated with several diseases (Licciulli et al. 2010b; Perez-Dominguez et al. 2021). For example, terminal myeloid differentiation is impaired by down-regulation of *PIR,* and reduction in pirin activity may be involved in differentiation arrest characteristic of acute myeloid leukemia (AML) (Licciulli et al. 2010a). Additionally, loss of pirin function may lead to immunodeficiency by hampering binding of p65 to DNA. Therefore, identification of the pathogenic variants in this protein can help in determining potential genetic factors leading to immunodeficiency and AML as well as other pathological conditions resulting for disruption of pirin function. Missense variants involving amino acid substitutions at functionally important sites may affect protein structure, stability, flexibility, ligand binding and protein–protein interactions (Zhang et al. 2012; Tamura et al. 2017; Hernandez and Facelli 2021; Birolo et al. 2021; Qi et al. 2021). Studying all variants experimentally is time-consuming, and demands significant resources and efforts. In silico analyses can ease the process by prioritizing missense variants for further experimental studies. In the present study, the impacts of pirin missense variants were explored using various in silico tools.

## Materials and methods

### Retrieval of missense variants of pirin and prediction of pathogenicity of the missense variants

The list of missense variants in pirin was retrieved from the Ensembl Genome Browser (Howe et al. 2021). The amino acid sequence of pirin was retrieved from UniProt Knowledgebase (UniProtKB) (Accession Number: O00625). Five different pathogenicity predicting tools—Sorting Intolerant From Tolerant (SIFT) (Sim et al. 2012), Polymorphism Phenotyping-2 (PolyPhen-2) (Adzhubei et al. 2010), PMut (López-Ferrando et al. 2017), Meta-SNP (Capriotti et al. 2013) and Rhapsody (Ponzoni et al. 2020) were used to predict the pathogenicity of these variants. Only the variants which were predicted to be harmful by all of these five tools were selected for further analyses.

### Determination of the effects of missense variants on the stability of pirin protein

First, 3D models of Fe^2+^ and Fe^3+^ bound conformations of pirin were generated by template-based modeling at SWISS-MODEL server (Waterhouse et al. 2018) using their X-ray crystallographic structures as templates (PDB IDs: 1J1L and 4GUL, respectively). These X-ray crystallographic structures were retrieved from the RCSB Protein Data bank (PDB) (Berman et al. 2000). These 3D structures were used as input in mutation Cutoff Scanning Matrix (mCSM) (Pires et al. 2014), DeepDDG (Cao et al. 2019), Impact of Nonsynonymous variations on Protein Stability 3D (INPS3D) (Savojardo et al. 2016), MAESTROweb (Laimer et al. 2015), and PremPS (Chen et al. 2020) to assess the destabilizing effects of the selected pirin variants.

### Structural analysis of pirin variants

The impacts of these variants on pirin structure was analyzed using Missense3D (Ittisoponpisan et al. 2019; Khanna et al. 2021). This is a structure database resource which can predict structural changes due to buried Gly replacement, Gly introduction in a bend, buried Pro introduction, Cys-Pro replacement, buried H-bond breakage, buried charge introduction, buried charge replacement, buried charge switch, buried salt bridge breakage, di-sulfide bond breakage, buried hydrophilic introduction, disallowed phi/psi introduction, cavity alteration, clash and secondary structure alteration. These changes may lead to the disruption of alpha helices or beta sheets, alterations in inter-residue interactions in the structures as well as unfavorable energy changes that affect the protein’s structure (Kajander et al. 2000; Betts and Russell 2003; Krieger et al. 2005; Ho and Brasseur 2005; Hubbard and Haider 2010; Chan et al. 2011).

### Assessment of the effects of variants on metal cofactor binding of pirin

To evaluate the impacts of the selected variants on pirin-iron interactions, the variant models were analyzed through metal ion-binding site prediction (MIB) (Lin et al. 2016) tool that uses fragment transformation method and can predict the metal-binding sites in a protein as well as perform metal ion docking (Lu et al. 2006).

### Determination of effects of missense variants of pirin on protein–protein interactions

The Fe^3+^ conformation of pirin modulates DNA binding by NF-κB p65 (Liu et al. 2013) and in a previous study, Ankyrin (ANK) repeat number 5, 6 and 7 of BCL3 was shown to bind to the Fe^2+^ conformation of pirin (Pang et al. 2004). Amino acid sequence of BCL3 covering these repeats (275–367) was retrieved from UniProtKB (Accession no. P20749) (Bateman et al. 2021). X-ray crystallographic structure obtained from RCSB PDB (PDB ID: 1K1A) was used as a template to generate a 3D model of these three repeat regions using SWISS-MODEL (Waterhouse et al. 2018). For p65 (UniProtKB Accession no. Q04207), a 3D model of its Rel homology domain (RHD) (19–306 amino acid residues) was prepared using the template 1RAM (PDB ID). Docking was performed using HDOCK (Yan et al. 2020) based on previously published docking results (Pang et al. 2004; Liu et al. 2013).

In case of docking with BCL3, the top model was superimposed on BCL3-p50 homodimer structure using UCSF Chimera 1.14 (Pettersen et al. 2021). Previously described method was followed for this superimposition, and our observation was consistent with the published data (Pang et al. 2004). Interactions between PIR-p65 in the top model were evaluated with Protein Interaction Calculator (PIC) web server (Tina et al. 2007). PIR-p65 interactions were consistent with the available information (Liu et al. 2013).

PIR-BCL3 and PIR-p65 complexes thus predicted were then utilized to determine the impacts of pirin missense variants on its interactions with partners using mCSM-PPI2 (Rodrigues et al. 2019), MutaBind2 (Zhang et al. 2020), SAAMBE-3D (Pahari et al. 2020) and BeAtMuSiC V1.0 (Dehouck et al. 2013).

### Simulation of the dynamics of pirin variants

To appraise the changes in protein dynamics caused by missense variants, the wild-type and mutant 3D models of pirin protein were subjected to simulation through a CABS coarse-grained protein model (Kmiecik et al. 2016). Previous study has suggested this method to be a proper alternative to conventional molecular dynamics that includes all atoms (Jamroz et al. 2013). Variants that showed significant negative impact in all the aforementioned analyses were selected for the simulation study. 3D models of variant structures were prepared using template-based modeling in SWISS-MODEL after manually substituting the wild-type residues with the mutant ones in the amino acid sequence of pirin. 3D models of both pirin conformations previously prepared with SWISS-MODEL were used as templates (Waterhouse et al. 2018). The dynamics of the wild-type and the variant structures were simulated with CABS-flex 2.0 using its default parameters (Kuriata et al. 2018).

### Haplotyping of the *PIR* variants

We were interested to see if any haplotype of *PIR* contains more than one missense variant. For this purpose, all SNPs with global minor allele frequencies > 0.001 were identified from dbSNP (Sherry et al. 2001). Haplotypes containing these polymorphisms and their frequencies were analyzed with the LDhap module of LDlink (Machiela and Chanock 2015). This module was used to calculate haplotype frequencies in five super-populations (African, Admixed American, East Asian, European and South Asian).

## Results

### Pathogenicity of the missense variants

A total of 153 missense variants of pirin were identified (Additional file 1: Table S1). The pathogenicity of these variants was predicted using five different tools (SIFT, PolyPhen2, PMut, Meta-SNP and Rhapsody). Each tool follows a different algorithm to predict pathogenicity. SIFT uses sequence homology-based approach for classifying a missense variant as either deleterious or tolerated (Sim et al. 2012). PolyPhen-2 classifies an amino acid substitution into probably damaging, possibly damaging, and benign on the basis of sequence, phylogenetic and structural characteristics of the substitution(Adzhubei et al. 2010, 2013). PMut uses neural networks to predict structure and evolutionary properties resulting from change in amino acid sequence (López-Ferrando et al. 2017). Rhapsody utilizes sequence coevolution data along with structure- and dynamics-based methods to predict pathogenicity of target variants (Ponzoni et al. 2020). Meta-SNP discriminates between disease-related and polymorphic nonsynonymous SNVs (nsSNV) through a random forest-based binary classification method and utilizes several different prediction tools to derive a consensus result (Capriotti et al. 2013). In this study, 45 variants were predicted to have harmful effects by all tools (Table 1). These variants were selected for further analyses.

**Table 1:** Pathogenic missense variants of pirin protein as predicted by five separate tools

Variant ID	Variants	SIFT	PolyPhen-2^a^	PMut	Meta-SNP	Rhapsody
rs372506134	G19A	Deleterious	Probably damaging	Disease	Disease	Deleterious
rs758349788	V24F	Deleterious	Probably damaging	Disease	Disease	Deleterious
rs1485928589	R25W	Deleterious	Possibly damaging	Disease	Disease	Deleterious
rs766252248	I28T	Deleterious	Probably damaging	Disease	Disease	Deleterious
rs746253345	P38L	Deleterious	Probably damaging	Disease	Disease	Deleterious
rs1277921319	D43H	Deleterious	Probably damaging	Disease	Disease	Deleterious
rs1356176104	H56Q	Deleterious	Probably damaging	Disease	Disease	Deleterious
rs780168534	H58R	Deleterious	Probably damaging	Disease	Disease	Deleterious
rs188288097	R59P	Deleterious	Probably damaging	Disease	Disease	Deleterious
rs188288097	R59Q	Deleterious	Probably damaging	Disease	Disease	Deleterious
rs1319331957	G60S	Deleterious	Probably damaging	Disease	Disease	Deleterious
rs1459166472	G60V	Deleterious	Probably damaging	Disease	Disease	Deleterious
rs780078643	G70A	Deleterious	Probably damaging	Disease	Disease	Deleterious
rs748257098	G70R	Deleterious	Probably damaging	Disease	Disease	Deleterious
rs780078643	G70V	Deleterious	Probably damaging	Disease	Disease	Deleterious
rs750390136	D77E	Deleterious	Probably damaging	Disease	Disease	Deleterious
rs1042818236	F78V	Deleterious	Probably damaging	Disease	Disease	Deleterious
rs757045955	H81P	Deleterious	Probably damaging	Disease	Disease	Deleterious
rs1464579620	G83D	Deleterious	Probably damaging	Disease	Disease	Deleterious
rs866898423	L90F	Deleterious	Probably damaging	Disease	Disease	Deleterious
rs149497039	A95V	Deleterious	Probably damaging	Disease	Disease	Deleterious
rs778749014	G98D	Deleterious	Probably damaging	Disease	Disease	Deleterious
rs140109164	G98S	Deleterious	Probably damaging	Disease	Disease	Deleterious
rs1467570812	H101Y	Deleterious	Probably damaging	Disease	Disease	Deleterious
rs752062795	Q115K	Deleterious	Probably damaging	Disease	Disease	Deleterious
rs1484554733	L116P	Deleterious	Probably damaging	Disease	Disease	Deleterious
rs1329364366	M126T	Deleterious	Probably damaging	Disease	Disease	Deleterious
rs1996173	P129L	Deleterious	Probably damaging	Disease	Disease	Deleterious
rs1294033379	V151D	Deleterious	Probably damaging	Disease	Disease	Deleterious
rs772251328	S161Y	Deleterious	Probably damaging	Disease	Disease	Deleterious
rs953093600	T167I	Deleterious	Possibly damaging	Disease	Disease	Deleterious
rs768193675	D173G	Deleterious	Probably damaging	Disease	Disease	Deleterious
rs780763035	D173N	Deleterious	Probably damaging	Disease	Disease	Deleterious
rs779413343	G179V	Deleterious	Possibly damaging	Disease	Disease	Deleterious
rs760795372	P187L	Deleterious	Possibly damaging	Disease	Disease	Prob.delet
rs1569195774	W190S	Deleterious	Possibly damaging	Disease	Disease	Deleterious
rs751833973	L220P	Deleterious	Probably damaging	Disease	Disease	Deleterious
rs1272804008	P245S	Deleterious	Probably damaging	Disease	Disease	Prob.delet
rs772771810	E248A	Deleterious	Probably damaging	Disease	Disease	Deleterious
rs769242287	E248D	Deleterious	Probably damaging	Disease	Disease	Deleterious
rs747391287	G254C	Deleterious	Probably damaging	Disease	Disease	Deleterious
rs996737505	G254V	Deleterious	Probably damaging	Disease	Disease	Deleterious
rs762648888	V257A	Deleterious	Probably damaging	Disease	Disease	Deleterious
rs764770692	M258I	Deleterious	Probably damaging	Disease	Disease	Deleterious
rs761242213	I264S	Deleterious	Probably damaging	Disease	Disease	Deleterious

^a^PolyPhen-2 classifies variants as benign, possibly damaging and probably damaging. Only those variants that were predicted to be “probably damaging” were considered to be harmful to increase the accuracy of prediction

### Destabilizing effects of the potentially pathogenic pirin variants

We used five different tools (mCSM, DeepDDG, INPS3D, MAESTROweb and PremPS) to evaluate the impacts of the variants on pirin stability. mCSM exploits the correlation between the impact of a mutation and atomic distance patterns surrounding the amino acid residue using graph-based signatures to predict stability changes (Pires et al. 2014). DeepDDG relies on neural network-based methods in the prediction of changes in protein stability due to point mutations (Cao et al. 2019). INPS3D uses descriptors to calculate ΔΔG values using a support vector regression (Savojardo et al. 2016). Descriptors extracted from the protein sequence to differentiate between wild and changed protein include a substitution score derived from the Blosum62 matrix, Kyte–Doolittle hydrophobicity scores of native and changed, the mutability index of the native residue, the molecular weights of native and changed residues, the difference in the alignment score between the native and variant sequences and an HMM, encoding evolutionary information of the wild-type sequence. MAESTROweb utilizes a multi-agent machine learning system based on protein structure to produce changes in unfolding free energy upon point mutation (Laimer et al. 2015). PremPS uses random forest regression scoring function to estimate effects of single mutations on protein stability. It employs an energy function to calculate unfolding Gibbs free energy (Chen et al. 2020).

The predicted changes in folding Gibbs free energy (ΔΔG) of both ferrous and ferric Pirin by these variants are listed in Table 2. ΔΔG value < − 1.0 kcal/mol (shown as bold) was considered to reflect destabilization and variants predicted by at least four tools to have ΔΔG value <  − 1.0 kcal/mol were considered to have significant destabilizing effects. Based on this criterion, nine variants identified to have significant destabilizing impacts on both conformations, whereas G98D may be more destabilizing for Fe^2+^ conformation as compared to the F^3+^ one.

**Table 2:** Impacts of potentially pathogenic missense variants on pirin stability

Variants^a,^	Changes in Fe^2+^ bound Pirin Stability, ∆∆G (kcal/mol)^b^					Variants^a,^	Changes in Fe^3+^ bound Pirin Stability, ∆∆G (kcal/mol)^b^				
	mCSM	DeepDDG	INPS3D	MAESTROweb	PremPS		mCSM	DeepDDG	INPS3D	MAESTROweb	PremPS
G19A	− 0.281	− **1.309**	0.12	− 0.045	− 0.85	G19A	− 0.348	− **1.419**	0.099	− 0.036	− 0.97
V24F	− **1.211**	− 0.729	− 0.38	0.736	− **1.19**	V24F	− **1.27**	− **1.047**	− 0.395	0.723	− **1.35**
R25W	− 0.567	− 0.445	− 0.574	0.396	− **1.27**	R25W	− 0.577	− 0.109	− 0.276	0.348	− **1.24**
***I28T***	− **2.411**	− **1.377**	− **2.543**	− **1.268**	− **2.03**	***I28T***	− **3.068**	− **1.927**	− **2.511**	− **1.317**	− **2.4**
P38L	− 0.932	− **3.204**	− 0.523	0.592	− 0.73	P38L	− 0.965	− **3.391**	− 0.523	0.426	− 0.73
D43H	− **1.98**	− 0.887	− 0.623	0.121	− 0.55	D43H	− **1.564**	− 0.509	− 0.679	0.212	− 0.71
H56Q	− 0.754	− 0.396	− 0.853	1	− **1.33**	H56Q	− 0.9	− 0.529	− 0.853	0.246	− **1.54**
H58R	− **1.891**	− **1.472**	− 0.258	− 0.005	− **1.51**	H58R	− **1.97**	− **1.444**	− 0.321	− 0.016	− **1.59**
***R59P***	− **1.594**	− **2.737**	− **1.567**	− **1.498**	− **1.41**	***R59P***	− **1.571**	− **2.829**	− **1.612**	− **1.767**	− **1.56**
***R59Q***	− **1.478**	− **1.578**	− **1.013**	− **1.66**	− **1.94**	***R59Q***	− **1.485**	− **1.538**	− **1.068**	− **1.781**	− **2.09**
G60S	− **1.841**	− **3.177**	− 0.912	− 0.479	− **1.56**	G60S	− **1.889**	− **3.076**	− 0.912	− 0.531	− **1.69**
G60V	− 0.605	− **3.761**	− **1.185**	0.065	− **1.44**	G60V	− 0.6	− **3.656**	− **1.185**	0.252	− **1.52**
G70A	− 0.78	− **2.001**	− 0.099	− 0.102	− 0.3	G70A	− 0.811	− **1.701**	− 0.125	− 0.014	− 0.4
G70R	− 0.746	− **1.566**	0.119	− 0.045	− 0.53	G70R	− 0.823	− **1.287**	0.227	− 0.027	− 0.62
G70V	− 0.685	− **2.375**	0.181	0.557	− 0.44	G70V	− 0.693	− **2.049**	0.21	0.218	− 0.52
D77E	− 0.601	− **1.97**	− 0.586	0.319	− 0.7	D77E	− 0.422	− **2.908**	− 0.65	0.328	− 0.77
**F78V**	− **1.476**	− **3.941**	− **2.152**	− 0.739	− **1.95**	***F78V***	− **1.549**	− **4.009**	− **2.086**	− **1.03**	− **2.23**
H81P	0.725	− **1.264**	− **1.286**	0.019	− 0.95	H81P	0.696	− **1.189**	− **1.242**	0.069	− **1.13**
G83D	− **1.452**	− **1.512**	− 0.683	0.599	− **1.27**	G83D	− **1.473**	− **1.529**	− 0.698	0.697	− **1.45**
L90F	− **1.604**	− **1.029**	− **1.032**	0.437	− **1.02**	L90F	− **1.585**	− 0.804	− **1.011**	0.441	− **1.16**
A95V	− 0.963	− **2.69**	− **1.356**	1.485	− **1.81**	A95V	− 0.899	− **2.24**	− **1.356**	1.541	− **1.9**
**G98D**	− **1.632**	− **3.5**	− 0.963	− **1.203**	− **1.64**	G98D	− **1.789**	− **3.266**	− 0.987	− 0.548	− **1.78**
G98S	− **1.299**	− **2.466**	− 0.93	0.106	− **1.31**	G98S	− **1.421**	− **2.281**	− 0.938	0.182	− **1.45**
H101Y	− 0.182	− 0.845	− 0.116	0.454	− **1.02**	H101Y	− 0.016	− 0.744	− 0.116	0.44	− **1.08**
Q115K	− 0.339	− **1.437**	− 0.796	− 0.556	− **1.46**	Q115K	− 0.263	− **1.195**	− 0.607	− 0.355	− **1.34**
***L116P***	− **1.323**	− **4.818**	− **3.54**	− **1.508**	− **2.79**	***L116P***	− **1.437**	− **4.762**	− **3.54**	− **1.645**	− **3.02**
M126T	− 0.114	− **1.028**	− **1.544**	− **1.123**	− **1.37**	M126T	− 0.145	− 0.798	− **1.533**	− **1.237**	− **1.27**
P129L	− 0.713	− **1.614**	− 0.579	0.436	− 0.69	P129L	− 0.748	− **1.719**	− 0.579	0.303	− 0.85
***V151D***	− **3.091**	− **5.314**	− **3.003**	− **1.201**	− **2.79**	***V151D***	− **3.117**	− **5.492**	− **3.003**	− **1.18**	− **2.94**
S161Y	− 0.62	− **1.719**	− 0.524	0.85	− 0.84	S161Y	− 0.607	− **1.933**	− 0.551	0.947	− 0.89
T167I	− 0.191	− 0.479	− 0.846	0.177	− **1.21**	T167I	− 0.158	− 0.518	− 0.846	0.512	− **1.21**
D173G	0.64	− **2.656**	− 0.949	− 0.026	0.01	D173G	0.482	− **2.415**	− **1.005**	− 0.021	− 0.09
D173N	− 0.137	− **1.544**	− 0.597	0.064	− 0.5	D173N	− 0.336	− **1.255**	− 0.742	0.141	− 0.48
G179V	− 0.612	− **2.029**	− **1.538**	− 0.93	− **1.44**	G179V	− 0.56	− **2.096**	− **1.542**	− 0.361	− **1.52**
P187L	− 0.369	− 0.886	− 0.814	0.066	− 0.78	P187L	− 0.343	− 0.91	− 0.814	0.037	− 0.85
***W190S***	− **2.767**	− **1.464**	− **2.712**	− **1.465**	− **1.26**	***W190S***	− **2.707**	− **1.415**	− **2.654**	− **1.568**	− **1.42**
***L220P***	− **1.386**	− **5.855**	− **3.385**	− **1.341**	− **2.32**	***L220P***	− **1.379**	− **5.872**	− **3.402**	− **1.261**	− **2.49**
**P245S**	− **1.457**	− **1.37**	− **1.046**	− 0.413	− **1.24**	**P245S**	− **1.554**	− **1.494**	− **1.046**	− 0.419	− **1.45**
E248A	− 0.689	− 0.603	− 0.581	− **1.544**	− 0.51	E248A	− 0.645	− 0.614	− 0.575	− **1.524**	− 0.67
E248D	− 0.685	− 0.853	− 0.729	− 0.862	− **1.03**	E248D	− 0.597	− 0.835	− 0.704	− 0.879	− **1.19**
G254C	− 0.606	− 0.619	− **1.685**	− 0.543	− **1.18**	G254C	− 0.511	− 0.649	− **1.636**	− 0.435	− **1.2**
G254V	− 0.199	− 0.862	− **1.482**	− 0.709	− **1.56**	G254V	− 0.149	− 0.94	− **1.4**	− 0.623	− **1.4**
***V257A***	− **2.006**	− **1.87**	− **2.659**	− **1.145**	− **2.1**	***V257A***	− **2.155**	− **1.948**	− **2.671**	− **1.143**	− **2.24**
M258I	− 0.971	− **1.677**	− **2.049**	0.268	− **1.67**	M258I	− 0.921	− **1.283**	− **2.079**	0.352	− **1.82**
***I264S***	− **2.812**	− **2.587**	− **3.226**	− **1.715**	− **2.56**	***I264S***	− **2.676**	− **2.499**	− **3.258**	− **1.757**	− **2.69**

^a^Variants that were predicted to significantly reduce pirin stability (∆∆G < − 1 kcal/mol) by all five tools are written in bold italics, whereas those that were predicted to have ∆∆G < − 1 kcal/mol by four tools are written in bold letters

^b^Negative ∆∆G values indicate decrease in pirin stability. ∆∆G values of < − 1 kcal/mol are written in bold letters, while ∆∆G values of > 1 kcal/mol are underlined

### Structural changes caused by the potentially pathogenic variants

Missense3D was used to predict the effects of the missense variants on pirin structure. First, 21 variants were predicted to have structural damages in the Fe^2+^ bound state. For the Fe^3+^ bound conformation, 20 variants were predicted to alter pirin conformation (Table 3). Six of the missense variants with bound Fe^2+^ (D43H, R59P, G60V, F78V, H101Y and D173G) were predicted to cause buried hydrogen breakage (Additional file 2: Table S2). Variants H101Y and D173G were predicted to alter cavity volume by more than 70 Å^3^ in both the Fe^2+^ and Fe^3+^ bound states. Besides, H58R and G83D were predicted to have altered cavities only in the Fe^3+^ bound conformation (Table 3).

**Table 3:** Effects of potentially pathogenic missense variants on pirin structure

**Variant**^**a**^	Fe2 + conformation	Variant^a^	Fe3 + conformation
	Structural Changes		Structural Changes
G19A	No structural damage detected	G19A	No structural damage detected
V24F	No structural damage detected	V24F	No structural damage detected
R25W	No structural damage detected	R25W	No structural damage detected
I28T	No structural damage detected	I28T	No structural damage detected
**P38L**	Cis pro replaced	**P38L**	This substitution triggers clash alert
**D43H**	Buried H-bond breakage, buried salt bridge breakage	**D43H**	Buried charge switch, Buried H-bond breakage, Buried salt bridge breakage
H56Q	No structural damage detected	H56Q	No structural damage detected
H58R	No structural damage detected	**H58R**	Cavity altered
**R59P**	Buried Pro-introduced, secondary structure altered, disallowed phi/psi, buried charge replaced, buried H-bond breakage, buried salt bridge breakage	**R59P**	Buried Pro introduced, secondary structure altered, disallowed phi/psi, buried charge replaced, buried H-bond breakage
**R59Q**	Buried charge replaced, buried salt bridge breakage	**R59Q**	Buried charge replaced, buried H-bond breakage
**G60S**	Buried Gly replaced, Gly in a bend	**G60S**	Disallowed phi/psi, buried Gly replaced, Gly in a bend
**G60V**	Buried Gly replaced, buried H-bond breakage, Gly in a bend	**G60V**	Clash, disallowed phi/psi, buried Gly replaced, buried H-bond breakage, Gly in a bend
**G70A**	Disallowed phi/psi, Gly in a bend	**G70A**	Clash, disallowed phi/psi, buried Gly replaced, buried H-bond breakage, Gly in a bend
**G70R**	Clash, disallowed phi/psi, Gly in a bend	**G70R**	Clash, disallowed phi/psi, Gly in a bend
**G70V**	Disallowed phi/psi, Gly in a bend	**G70V**	Disallowed phi/psi, Gly in a bend
D77E	No structural damage detected	D77E	No structural damage detected
**F78V**	Buried H-bond breakage	F78V	No structural damage detected
H81P	No structural damage detected	H81P	No structural damage detected
G83D	No structural damage detected	**G83D**	Cavity altered
L90F	No structural damage detected	L90F	No structural damage detected
A95V	No structural damage detected	A95V	No structural damage detected
**G98D**	Buried charge introduced, disallowed phi/psi, buried Gly replaced	**G98D**	Buried charge introduced, disallowed phi/psi, buried Gly replaced
**G98S**	Disallowed phi/psi, buried Gly replaced	**G98S**	Disallowed phi/psi, buried Gly replaced
**H101Y**	Buried charge replaced, buried H-bond breakage, cavity altered	**H101Y**	Buried charge replaced, buried H-bond breakage, cavity altered
Q115K	No structural damage detected	Q115K	No structural damage detected
**L116P**	Buried Pro introduced, Disallowed phi/psi	**L116P**	Buried Pro introduced, disallowed phi/psi
M126T	No structural damage detected	M126T	No structural damage detected
P129L	No structural damage detected	P129L	No structural damage detected
**V151D**	Buried hydrophilic introduced, buried charge introduced	**V151D**	Buried hydrophilic introduced, buried charge introduced
S161Y	No structural damage detected	S161Y	No structural damage detected
T167I	No structural damage detected	T167I	No structural damage detected
**D173G**	Buried H-bond breakage Cavity altered, buried / exposed switch	**D173G**	Buried H-bond breakage, cavity altered, buried / exposed switch
**D173N**	Buried charge replaced	D173N	No structural damage detected
G179V	No structural damage detected	G179V	No structural damage detected
P187L	No structural damage detected	P187L	No structural damage detected
W190S	No structural damage detected	W190S	No structural damage detected
**L220P**	Buried Pro introduced	**L220P**	Buried Pro introduced
P245S	No structural damage detected	P245S	No structural damage detected
E248A	No structural damage detected	E248A	No structural damage detected
E248D	No structural damage detected	**G254C**	Disallowed phi/psi
**G254C**	Disallowed phi/psi	**G254V**	Disallowed phi/psi
**G254V**	Disallowed phi/psi	E248D	No structural damage detected
V257A	No structural damage detected	V257A	No structural damage detected
M258I	No structural damage detected	M258I	No structural damage detected
I264S	No structural damage detected	I264S	No structural damage detected

^a^Variants that were predicted to be damaging to pirin structure are written in bold letters

### Effects of variants on metal cofactor binding

Since the iron-binding site of pirin is crucial for its biological activity, the MIB tool (Lin et al. 2016) was used to investigate the effects of missense variants of pirin on iron binding. This tool uses fragment transformation method for binding site prediction and docking of metal ions. The overall binding scores of Fe^2+^ to pirin protein were similar for wild-type structure and the missense variant, except H56Q, H58R and H101Y (Additional file 3: Table S3). This pattern was observed for Fe^3+^, too. Residues His-56, His-58 and His-101 are three of four residues reported in a previous study to be the constituents of metal-binding site (Pang et al. 2004).

### Effects of missense variants on protein–protein interactions

**Fig. 1:**
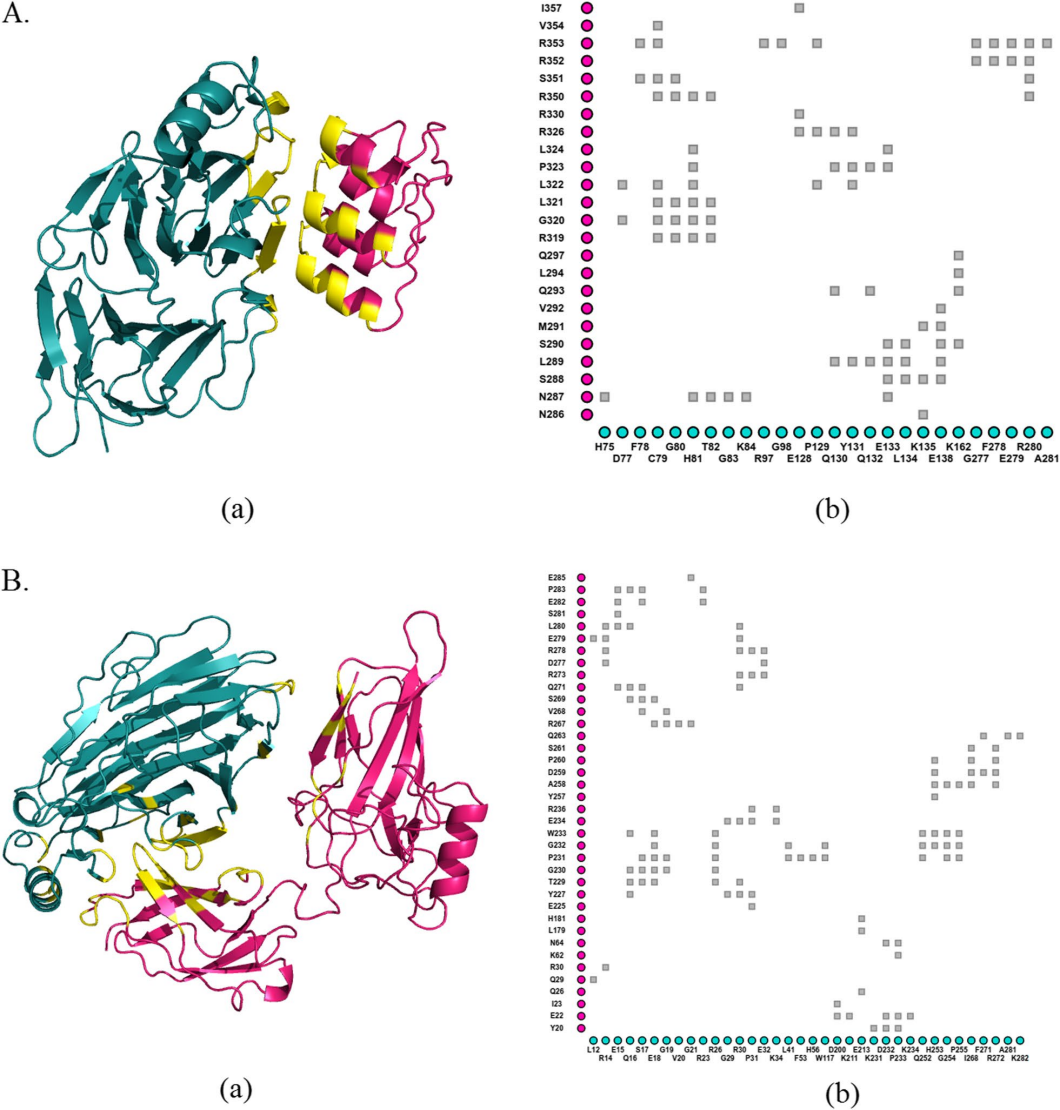
**Interactions between pirin and its binding partners.** Docking was performed between Fe^2+^ conformation of pirin and three ankyrin repeat domains (ANK 5-7) of BCL3 (**A**), as well as, Fe^3+^ conformation of pirin and Rel homology domain (RHD) of p65 (residues 19-306) (**B**). The protein–protein complex structures were depicted using PyMOL (Schrodinger and Delano 2020) (Aa and Ba). Pirin is illustrated in deep teal and its partners in hot pink. Protein–protein interfaces are delineated in yellow. Inter-residue interactions are delineated with iCn3D (Wang et al. 2020) (Ab and Bb). Each gray square represents contacts/interactions within 6 (Å). Residues of pirin are displayed in the x-axis and those of its binding partners are in the y-axis

After generation of models for BCL3 and p65 through template-based modeling using SWISS-MODEL, pirin was docked to each of these proteins using HDOCK (Fig. 1) (Yan et al. 2020). Interactions of pirin with BCL3 and p65 were then analyzed for the impacts of missense variants in pirin using mCSM-PPI2 (Rodrigues et al. 2019), MutaBind2 (Zhang et al. 2020), SAAMBE-3D (Pahari et al. 2020) and BeAtMuSiC V1.0 (Dehouck et al. 2013). mCSM-PPI2 predicts the effects of missense variants on protein–protein binding affinity by concentrating on the inter-residue non-covalent interaction network using optimized graph-based signatures like graph kernels, evolutionary information, complex network metrics and energetic terms (Rodrigues et al. 2019). MutaBind2 exploits molecular mechanics force fields, statistical potentials and fast side-chain optimization algorithms built via random forest method (Zhang et al. 2020). SAAMBE-3D uses machine learning (Pahari et al. 2020). BeAtMuSiC V1.0 utilizes a set of statistical potentials derived from known protein structures and combines the effect of the mutation on the strength of the interactions at the interface, and on the overall stability of the complex (Dehouck et al. 2013).

In this study, negative values for ∆∆G indicated reduction in protein–protein binding affinity, and variants with predicted ∆∆G < -− 1 kcal/mole (by at least three tools) were considered to significantly decrease such affinity. Four variants (R59P, F78V, H81P and L116P) in case of pirin-BCL3 were found to be substantially destabilizing in case of pirin-BCL3 complex (Table 4). However, no variant had such considerable effect on pirin-p65 interactions. Interestingly, several variants (G98D, P129L, W190S and L220P in case of pir-BCL3 complex, and R59P, L116P and G254C in case of pirin-p65 complex) were predicted to have ∆∆G < − 1 kcal/mol by two tools and < -− 0.85 kcal/mol by at least one of the remaining two tools (Table 4). Such variants might also have significant destabilizing effects.

**Table 4:** Effects of potentially pathogenic missense variants on stability of pirin-BCL3 and pirin-p65 interactions

Variants^a^	Changes in pirin-BCL3 complex stability, ∆∆G (kcal/mol)^b^				Variants^a^	Changes in pirin/p65 complex stability, ∆∆G (kcal/mol)^b^			
	mCSM-PPI2	MutaBind2	SAAMBE3D	BeAtMuSic V1.0		mCSM-PPI2	MutaBind2	SAAMBE3D	BeAtMuSiC V1.0
G19A	− 0.119	− *1*	− 0.1	− 0.25	*G19A*	− *0.881*	− *0.88*	− 0.43	− **1.29**
V24F	0.229	− *0.98*	0.29	− 0.08	V24F	0.407	− 0.66	0.03	− 0.2
R25W	− 0.171	− 0.43	− 0.34	− 0.22	R25W	0.019	− 0.26	− 0.34	− 0.05
I28T	− 0.195	− 0.74	0.5	− *0.84*	I28T	− 0.19	− 0.64	0.5	− *0.93*
P38L	− 0.51	− *0.93*	0.1	− 0.58	P38L	− 0.44	− 0.57	0.1	− 0.41
D43H	− 0.11	− 0.63	− 0.07	0.09	D43H	0.171	− **1.09**	− 0.15	0.15
H56Q	− 0.339	− 0.47	− 0.15	− 0.31	H56Q	− 0.459	− 0.46	− 0.38	− 0.47
H58R	− 0.015	− *0.9*	− 0.18	− 0.34	H58R	− 0.055	− 0.39	− 0.29	− 0.44
***R59P***	− **1.139**	− **1.15**	− **1.9**	− **1.1**	**R59P**	− **1.102**	− 0.66	− **1.9**	− *0.97*
R59Q	− 0.656	− 0.74	− 0.72	− 0.65	R59Q	− 0.462	− 0.3	− 0.72	− 0.48
G60S	− 0.673	− *0.99*	0.15	− 0.6	G60S	− 0.438	− 0.42	0.15	− 0.59
G60V	− *0.999*	− **1.85**	− 0.16	− 0.46	G60V	− *0.866*	− 0.74	− 0.16	− 0.46
G70A	− 0.114	− 0.46	0	− *0.9*	G70A	− 0.017	− 0.25	0	− 0.67
G70R	− 0.309	− 0.31	− 0.34	− *0.92*	G70R	− 0.248	− 0.08	− 0.34	− 0.81
G70V	− 0.43	− 0.54	− 0.16	− 0.71	G70V	− 0.308	0	− 0.16	− 0.68
D77E	− 0.668	− **1.3**	− 0.7	− *0.95*	D77E	− 0.059	− 0.62	− 0.6	− 0.65
***F78V***	− **1.472**	− **2.3**	− **1.16**	− *0.99*	F78V	− 0.47	− 0.36	− *0.85*	− 0.7
***H81P***	− **2.528**	− **2.97**	− **1.57**	− **1.86**	H81P	− 0.674	− 0.49	− 0.79	− 0.81
G83D	− 0.286	− **1.74**	− 0.61	− *0.87*	G83D	0.167	− 0.49	0.03	− 0.43
L90F	0.712	− *0.95*	− 0.03	− 0.06	L90F	0.401	− 0.73	− 0.03	− 0.13
A95V	0.12	− 0.78	0.43	0.02	A95V	0.207	− 0.77	0.43	− 0.09
**G98D**	− *0.959*	− **1.84**	0.05	− **1.47**	G98D	− 0.464	− 0.54	0.05	− **1.3**
G98S	− *0.938*	− *0.82*	0.04	− **1.18**	G98S	− 0.512	− 0.66	0.04	− **1.09**
H101Y	− 0.336	− **1.1**	− 0.72	− 0.06	H101Y	− 0.077	− 0.83	− 0.72	− 0.09
Q115K	− 0.452	− 0.49	− 0.26	− 0.47	Q115K	− 0.322	− 0.38	− 0.26	− 0.41
***L116P***	− **1.048**	− **1.3**	− **1.11**	− **1.71**	**L116P**	− *0.875*	− 0.61	− **1.11**	− **1.91**
M126T	− 0.026	− 0.25	− 0.35	− 0.14	M126T	− 0.222	− 0.43	− 0.35	− 0.02
**P129L**	− **1.058**	− **1.19**	0.13	− *0.99*	P129L	− 0.083	− 0.48	0.28	− 0.1
V151D	− 0.418	− *0.81*	− 0.15	− **1.05**	V151D	− 0.364	− 0.63	− 0.15	− **1.17**
S161Y	− 0.079	− **1.26**	0.13	− 0.18	S161Y	0.142	− 0.5	0.13	− 0.24
T167I	− 0.279	− **1.02**	0.03	− 0.15	T167I	− 0.154	− 0.43	0.03	− 0.09
D173G	− 0.5	− **1.1**	− 0.5	− 0.34	D173G	− 0.38	− 0.42	− 0.5	− 0.38
D173N	− 0.608	− 0.52	− **1.24**	− 0.05	D173N	− 0.792	− 0.71	− **1.24**	− 0.13
G179V	− 0.02	− *0.92*	− 0.23	− 0.61	G179V	− 0.075	− 0.38	− 0.23	− 0.57
P187L	− 0.248	− 0.46	0.13	− 0.39	P187L	− 0.23	− 0.4	0.13	− 0.45
**W190S**	− 0.117	− **1.19**	− **1.19**	− *0.99*	W190S	− 0.21	− 0.51	− **1.19**	− *0.9*
**L220P**	− *0.974*	− **1.22**	− *0.84*	− **1.15**	L220P	− *0.931*	− 0.45	− 0.84	− **1.11**
P245S	− 0.16	− *0.88*	0.15	− 0.5	P245S	− 0.131	− 0.51	0.15	− 0.53
E248A	− 0.118	− 0.24	− 0.32	− 0.38	E248A	− 0.157	− 0.17	− 0.32	− 0.3
E248D	− 0.003	− 0.46	− 0.51	− 0.27	E248D	0.042	− 0.21	− 0.51	− 0.45
G254C	− 0.153	− 0.61	0.03	− 0.48	**G254C**	− *0.897*	− **1.22**	− 0.03	− **1.08**
G254V	− 0.079	− *0.95*	− 0.09	− 0.72	G254V	− 0.62	− *0.95*	− 0.56	− **1.55**
V257A	− 0.316	− *0.99*	− 0.37	− **1.04**	V257A	− 0.324	− 0.69	− 0.49	− **1.14**
M258I	− 0.357	− *0.9*	− 0.23	− 0.07	M258I	− 0.387	− **1.06**	− 0.23	− 0.15
I264S	− 0.301	− *0.95*	0.08	− *0.98*	I264S	− 0.28	− 0.49	0.08	− *0.9*

^a^Variants that were predicted to significantly reduce protein–protein binding affinity (∆∆G < − 1 kcal/mol) by at least three tools are written in bold italics. Variants with predicted ∆∆G < − 1 kcal/mol by two tools and with predicted ∆∆G values between − 0.85 and − 1 kcal/mol by at least one tool are written in bold letters

^b^Negative ∆∆G values indicate decrease in protein–protein binding affinity. ∆∆G values of < − 1 kcal/mol are written in bold letters, while ∆∆G values between − 0.85 and − 1 kcal/mol are written in italics

### Alterations in the dynamics of pirin

**Fig. 2:**
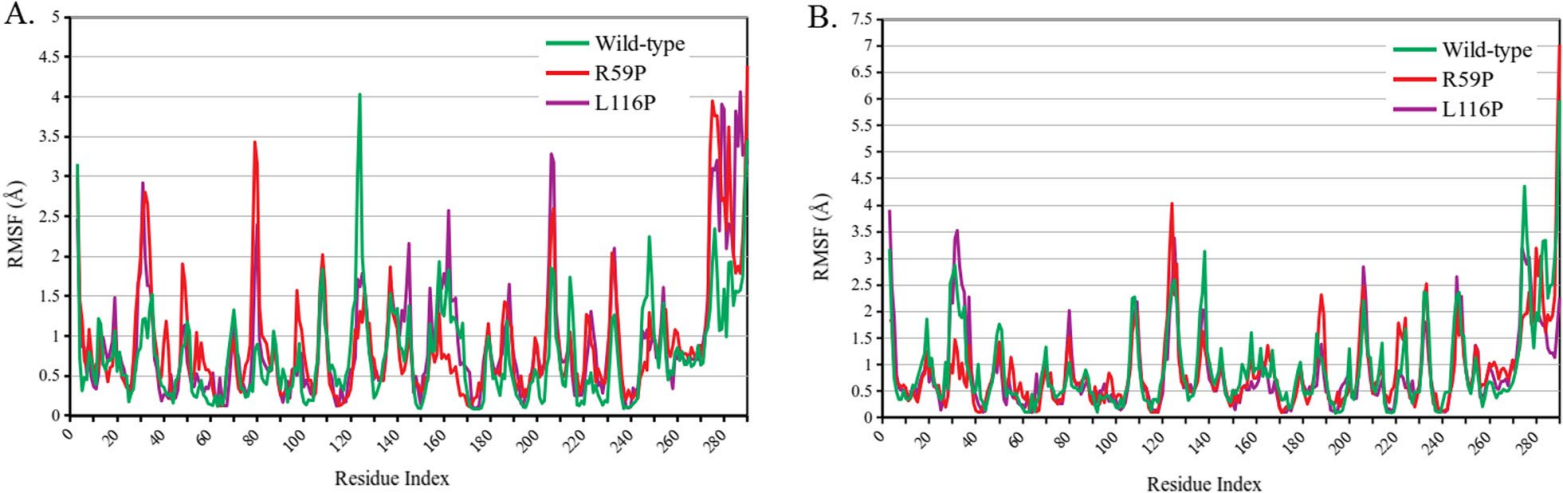
**Fluctuation profiles of the wild-type and two variant (R59P and L116P) structures of pirin.** A comparison between the predicted residue fluctuation profiles of the wild-type and the variant structures in case of Fe^2+^ (**A**) and Fe^3+^ (**B**) conformations of pirin is graphically presented. RMSF denoting root mean square fluctuation in Å is displayed in the y-axis and pirin residues are displayed in the x-axis

Two variants (R59P and L116P) were found to destabilize pirin (Table 2), damage its structure (Table 3) and diminish pirin’s binding affinity to its partners (Table 4). Protein dynamics simulation was performed for these two variant structures, along with the wild-type ones, in both Fe^2+^ and Fe^3+^ bound conformations. Residue fluctuation profile revealed three areas with major alterations in fluctuation (Fig. 2). These areas are roughly from residue 28 to 37, 78 to 84, 120 to 125. The first and third regions were found to have altered fluctuation for both variants in both conformations. However, the second region, from78 to 84, showed large alteration for Fe^2+^ bond conformation only. Region of amino acids 28 to 37 overlap the R-shaped region responsible for p65 binding. 78 to 84 and 120 to 125 amino acid residues overlap two of the acidic patches responsible for BCL3 binding (Fig. 1). There are other regions for each of the two variants that show altered fluctuation but the two conserved iron-binding clusters show no major fluctuation in any case.

### Haplotyping of the variants

With the goal of identifying haplotypes containing more than one pirin missense variants, haplotype frequencies in five super-populations were calculated (Table 5). Three haplotypes were found to harbor two variant alleles. Each of these haplotypes however contained rs8094T allele, which is synonymous.

**Table 5:** Frequencies of haplotypes containing pirin variants

SNP IDs	Haplotypes^a^	Haplotype frequencies					
		ALL	African	American	East Asian	European	South Asian
rs75378219_rs34104000_rs8094_rs35715407_rs34149789	C_A_C_C_C	0.5272	0.5902	0.4828	0.5314	0.4295	0.571
	C_A_**T**_C_C	0.4286	0.2602	0.4847	0.4686	0.5705	0.429
	C_**G**_C_C_C	0.0238	0.0788	0.021	–	–	–
	**T**_A_C_C_C	0.0056	0.0189	0.0038	–	–	–
	*C_A_****T****_C_****G***	0.0045	0.016	0.0019	–	–	–
	C_A_C_**A**_C	0.0029	0.011	–	–	–	–
	***T****_A_****T****_C_C*	0.0029	0.01	0.0019	–	–	–
	C_A_C_C_G	0.0024	0.007	0.0038	–	–	–
	*C_****G****_****T****_C_C*	0.0021	0.008	–	–	–	–

^a^Variant DNA bases are written in bold letters, and haplotypes containing more than one variant are written in italics

## Discussion

The present study aimed at prioritizing pathogenic missense variants of pirin protein for further experimental analysis. For this purpose, out of 153 missense variants or pirin, 45 were selected for further analyses as they were uniformly predicted to be pathogenic (Table 1). The impacts of these selected variants on pirin’s stability (Table 2), structure (Table 3), cofactor binding (Additional file 3: Table S3) and interactions with binding partners (Table 4) were predicted using multiple in silico tools to enhance prediction accuracy. Additionally, two variant structures (R59P and L116P) were subjected to protein dynamics simulation (Fig. 2) due to their predicted substantial effects on pirin structure and possibly function. Our findings indicate that these two variants, along with five other variants (R59Q, F78V, G98D, V151D and L220P), should be subjected to further experimental investigations.

Two domains of pirin are the N-terminal domain spanning residues 3–134 and the C-terminal domain comprising residues 143–290 (Pang et al. 2004). Sequence of the N-terminal domain has remained significantly conserved in mammals, plants and prokaryotes, notably in two regions spanning residues 52–70 (cluster 1) and residues 88–106 (cluster 2) (Wendler et al. 1997). Together these regions contain a total of four metal coordinating residues that are strictly conserved among species. N-terminal domain and iron-binding residues are thus important for pirin function. R59P lie in the conserved cluster 1 and L116P is close to conserved cluster 2, both residues located in the N-terminal. The C-terminal domain contain neither any metal-binding site nor conserved residues (Pang et al. 2004).

Earlier studies reported that pirin interacts with BCL3 and p65 which are members of the IκB and NF-κB family, respectively (Dechend et al. 1999; Liu et al. 2013). A large acidic patch with residues 77–82, 97–103, and 124–128 is present on the surface of pirin N-terminal domain. This patch has been shown to interact with the large basic patch on ankyrin repeats 6 and 7 of BCL3, whereas residues in C-terminal domain may interact with ankyrin repeat 5 (Pang et al. 2004). An R-shaped region is a notable pirin surface region which directly interacts with p65, comprising most of the central binding surface between them (Liu et al. 2013). It is made up of the residues 7–41 and 53–62, which also spans the surrounding area of the metal-binding cavity at the N-terminus (Liu et al. 2013). Arg59 lies in this R-shaped region needed for p65 binding. On the other hand, Leu116 is located in between two acidic patches on the surface of pirin required for interaction with BCL3. Therefore, R59P and L116P are likely to have significant impacts on pirin functionalities.

Residue fluctuation profiles indicated both R59P and L116P cause significant fluctuation in amino acid residues 28 to 37, which is part of the R-shaped region responsible for p65 binding. The two variants also alter fluctuation in regions of 78 to 84 and 120 to 125 amino acid residues which overlap the acidic patches of residues 77 to 82 and 124 to 128 (Fig. 2). Based on previously reported roles of R-shaped region and acidic patch in interaction of pirin with P65 and BCL3 (Pang et al. 2004; Liu et al. 2013), it can be stated that the altered fluctuation due to these variants might hamper the protein–protein interactions of pirin. Our findings are consistent with such assumptions (Table 4).

The metal-binding pocket is situated in the N-terminal domain of pirin and contains residues His-56, His-58, His-101 and Glu-103 which coordinate with ferrous/ferric ion. Metal binding has been reported to be crucial for interactions between pirin and its binding partners. A small-molecule inhibitor TPh A inserts into the Fe^2+^ containing pocket and prevents the cellular activity of pirin by disrupting the formation of pirin-Bcl3 complex (Miyazaki et al. 2010). Moreover, the binding of Fe^3+^ instead of Fe^2+^ alters pirin conformation and thus helps it to bind to p65 of NF-κB family. This iron center plays a role in the allosteric control of the R-shaped surface loop region (Liu et al. 2013). Pirin may therefore stimulate gene expression by BCL3.-p50 complex and p65 in the Fe^2+^ and Fe^3+^ bound states, respectively. Fe^2+^ to Fe^3+^ conversion occurs as the nuclear environment becomes more oxidizing. Hence, the activity of pirin and its related gene expression is dependent on the redox state. The iron-binding center contributes significantly in this process. Since these interactions can be disrupted by the aforementioned missense variants, pathways of the immune system and cell division, modulated by BCL3 and p65 can thus be adversely impacted, leading to disease conditions.

One of the variants that alter the metal-binding residues, H101Y, was found to cause structural damage (Table 3). In addition, the H101Y variant replaces the buried charge, breaks a buried H-bond and alters cavity by 70 Å, in both Fe^2+^ and Fe^3+^ bound conformations (Table 3 and Additional file 2: Table S2). Only in the Fe^3+^ bound state, the variant H58R, another iron coordinating residue, was predicted to cause an alteration in the cavity. In the metal-binding analysis, variants H56Q, H58R and H101Y had scores inconsistent with the wild-type, which may be suggestive of the possible effects of these variants on pirin–iron associations.

L220P in the less conserved C-terminal domain was predicted to alter stability and structure of both Fe^2+^ and Fe^3+^ bound conformations (Table 2 and 3). It may also interfere with interactions with BCL3, but not with p65 (Table 4).

Two other variants of pirin (F78V and G98D) are of considerable interest on the basis of our findings. The F78V variant was found to break buried H-bond in Fe^2+^ bound state only (Additional file 2: Table S2), but it was predicted to destabilize both Fe^2+^ and Fe^3+^ bond conformations. It is part of the acidic patches that bind with ARD of BCL3. In agreement with this information, our study predicted the F78V variant to hamper binding of pirin with BCL3. No such relationship has been observed for p65. In the case of variant G98D, introduction of buried charge and replacement of glycine create structural changes in Fe^2+^ as well as Fe^3+^ bound conformations (Table 3). Destabilization of both conformations and hindrance in interaction with BCL3 is significant for this variant (Tables 2 and 4).

In spite of having no effect on protein–protein interactions, variants R59Q and V151D draw attention as both of these significantly destabilize and alter structures for the Fe^2+^ and Fe^3+^ bound conformations (Table 2 and 3). Four variants (I28T, W190S, V257A and I264S) were identified to significantly destabilize the protein. However, no effect on structure or protein–protein interaction was predicted. In contrast, P38L, D43H, H58R, G60S, G60V, G70A, G70R, G70V, G98S, H101Y, D173G, D173N, G254C and G254V were found to cause structural damages whereas the stability of those structures was predicted to be unaffected. Here, buried hydrogen bonds were disrupted by D43H, R59P, G60V and D173G (Additional file 2: Table S2). On the other hand, most of the variants displayed similar results regarding redox state of iron, showing damages in both conformations. Strikingly, D173N and H58R were exceptions. Structural damage has been predicted for D173N only when bound to Fe^2+^ and for H58R only when bound to Fe^3+.^ This phenomenon and the differential roles of Asp-173 and His-58 in two pirin conformations should be clarified in future studies.

Although variants P38L, H58R, G60S and G60V are located in the R-shaped region responsible for p65 binding, these residues did not appear to exert any effect on protein–protein interactions (Table 4). The absence of their direct interactions with p65 might explain these results (Fig. 1). His-58 and His-101 are two of the iron coordinating residues and variants at these sites (H58R and H101Y) were found to minimize the binding affinity of iron compared to wild-type protein. According to MIB tool prediction, variants other than the four metal coordinating ones were found to show no adverse effect in iron binding (Additional file 3: Table S3).

Another variant can be of consideration. H81P has been reported by four tools to disrupt interaction with BCL3. Although it has no other negative effect, being part of the acidic patch makes it a significant residue for BCL3 interaction and functionality of pirin.

Absence of any haplotype with more than one missense variant (Table 5) indicates that the presence of multiple missense variants in the same individual is unlikely. So, exploring the combinatorial effect of more than one missense variant in pirin protein may not be necessary, and identifying individual effects of missense variants may be sufficient in this connection.

It should be noted that a recent study identified V257A, I28T and I264S variants to have significant destabilizing effects on pirin structure (Suleman et al. 2021). Our study also found these variants to substantially reduce the stability of both pirin conformations (Table 2). However, the previous study had a lower number of initial missense variants (119, as compared to 153 in our study), and chose less variants for further analysis (24, as compared to 45 in our study). Besides, impacts of variants on pirin structure, cofactor binding and interactions with other proteins were not elaborated. Furthermore, the aforementioned study did not differentiate between the two conformations of pirin. Therefore, our study appears to be the most comprehensive exploration of the effects of pirin missense variants so far. The variants identified in this study for further experimental clarifications thus contribute to the existing list of prioritized pirin variants.

## Conclusions

Since pirin plays a crucial role in regulation of multiple gene expressions, variants that alter its structure and impede its functions can contribute to the pathogenesis of various diseases. Prioritizing these variants for further experimentation is therefore essential. In the present study, we used multiple in silico tools to assess the possible pathogenicity of a total of 153 missense variants and appraise the impacts of a selected set of variants on pirin’s structure and functions. Based on our findings, we propose that seven variants (R59P, L116P, L220P, F78V, G98D, R59Q and V151D) should be considered for further investigations. In addition, four other variants (H58R, H101Y, D173N and H81P) can also be important targets of analysis. Since haplotypes with more than one pirin missense variant could not be found, exploring effects of individual variants should be enough for identifying roles of variants in disrupting pirin’s functions. Our findings thus significantly contribute to the existing knowledge regarding pathogenic variants of pirin. Future studies should focus on the possibility of using these variants as disease biomarkers.

## Supplementary Information

**Additional file 1**: Table S1. List of missense variants of pirin.

**Additional file 2**: Table S2. Buried hydrogen bonds broken by pirin missense variants.

**Additional file 3**: Table S3. Effects of missense variants on metal binding of pirin according to MIB tool.

## Data Availability

All data generated or analyzed during this study are included in this published article (and its Additional files).

## Abbreviations

NFI/CTF1: Nuclear factor I/CCAAT box transcription factor
NF- κB: Nuclear factor κB
I κB: Inhibitor of kappa-B
BCL3: B-cell lymphoma 3-encoded protein
Nrf2: Nuclear factor erythroid-derived 2-like 2
ARE: Antioxidant response elements
AML: Acute myeloid leukemia
SNP: Single nucleotide polymorphism
nsSNV: Nonsynonymous single nucleotide variants
