# Supplementary material for: A comprehensive in silico exploration of the impacts of missense variants on two different conformations of human pirin protein
Source: Bull Natl Res Cent. 2022 Jul 30;46(1):225. doi: 10.1186/s42269-022-00917-7 (PMC9362109; doi:10.1186/s42269-022-00917-7)
Supplement: Supplementary file 1 — Additional file 1: Table S1. List of missense variants of pirin. [file 42269_2022_917_MOESM1_ESM.docx]

**Supplementary Table 1:** List of missense variants of pirin

| **Variant ID** | **Location** | **Alleles** | **Amino Acid Substitution** | **Amino Acid Coordinates** |
| --- | --- | --- | --- | --- |
| rs758165582 | X:15385014 | A/G | I/T | 288 |
| rs1569190194 | X:15385029 | G/A | T/I | 283 |
| rs200265489 | X:15385030 | T/A/C | T/S | 283 |
| rs200265489 | X:15385030 | T/A/C | T/A | 283 |
| rs1569190207 | X:15385036 | C/G | A/P | 281 |
| rs749390761 | X:15385038 | C/T | R/K | 280 |
| rs770992195 | X:15385056 | G/T | A/E | 274 |
| rs779037264 | X:15385057 | C/T | A/T | 274 |
| rs772371663 | X:15385060 | T/C | N/D | 273 |
| rs1412757910 | X:15385074 | A/G | I/T | 268 |
| rs376249765 | X:15385083 | G/A/C | S/F | 265 |
| rs376249765 | X:15385083 | G/A/C | S/C | 265 |
| rs761242213 | X:15385086 | A/C | I/S | 264 |
| rs1361734602 | X:15385087 | T/C | I/V | 264 |
| rs141898566 | X:15385089 | T/C | E/G | 263 |
| rs146291069 | X:15385095 | T/C | N/S | 261 |
| rs1442915375 | X:15385098 | G/T | T/N | 260 |
| rs1329224945 | X:15385101 | T/C | N/S | 259 |
| rs764770692 | X:15385103 | C/G | M/I | 258 |
| rs750089812 | X:15385105 | T/C | M/V | 258 |
| rs762648888 | X:15385107 | A/G | V/A | 257 |
| rs996737505 | X:15385116 | C/A | G/V | 254 |
| rs747391287 | X:15390185 | C/A | G/C | 254 |
| rs768962942 | X:15390187 | T/C | H/R | 253 |
| rs769242287 | X:15390201 | T/G | E/D | 248 |
| rs772771810 | X:15390202 | T/G | E/A | 248 |
| rs1272804008 | X:15390212 | G/A | P/S | 245 |
| rs944737824 | X:15390235 | T/A | H/L | 237 |
| rs751207133 | X:15390241 | C/T | R/K | 235 |
| rs759434052 | X:15390245 | T/C | K/E | 234 |
| rs75378219 | X:15390251 | C/T | D/N | 232 |
| rs34104000 | X:15397459 | A/G | V/A | 228 |
| rs8094 | X:15397461 | C/A/G/T | Q/H | 227 |
| rs8094 | X:15397461 | C/A/G/T | Q/H | 227 |
| rs1436772569 | X:15397466 | C/G | V/L | 226 |
| rs758506659 | X:15397469 | T/C | S/G | 225 |
| rs1382486023 | X:15397472 | C/G | D/H | 224 |
| rs148750447 | X:15397474 | C/T | G/D | 223 |
| rs1322780285 | X:15397475 | C/T | G/S | 223 |
| rs1403604570 | X:15397480 | C/T | G/E | 221 |
| rs751833973 | X:15397483 | A/G | L/P | 220 |
| rs142402391 | X:15397502 | G/C/T | P/A | 214 |
| rs142402391 | X:15397502 | G/C/T | P/T | 214 |
| rs373762633 | X:15397505 | C/G | E/Q | 213 |
| rs756384418 | X:15397508 | T/C | I/V | 212 |
| rs1010665693 | X:15397526 | C/T | D/N | 206 |
| rs754988948 | X:15407517 | T/A | D/V | 200 |
| rs767802108 | X:15407524 | A/G | S/P | 198 |
| rs1343063780 | X:15407527 | T/C | I/V | 197 |
| rs200350682 | X:15407529 | G/A | T/M | 196 |
| rs1569195774 | X:15407547 | C/G | W/S | 190 |
| rs753011154 | X:15425907 | T/G | K/N | 188 |
| rs760795372 | X:15425911 | G/A/C | P/L | 187 |
| rs760795372 | X:15425911 | G/A/C | P/R | 187 |
| rs764587964 | X:15425912 | G/A | P/S | 187 |
| rs754259162 | X:15425915 | T/C | I/V | 186 |
| rs750922769 | X:15425927 | G/C | H/D | 182 |
| rs757971715 | X:15425932 | G/A | A/V | 180 |
| rs779413343 | X:15425935 | C/A | G/V | 179 |
| rs1430906408 | X:15425942 | C/A | D/Y | 177 |
| rs768193675 | X:15425953 | T/C | D/G | 173 |
| rs780763035 | X:15425954 | C/T | D/N | 173 |
| rs953093600 | X:15425971 | G/A | T/I | 167 |
| rs1344282551 | X:15425972 | T/A | T/S | 167 |
| rs773172117 | X:15425974 | C/T | R/H | 166 |
| rs762777791 | X:15425975 | G/A/T | R/C | 166 |
| rs762777791 | X:15425975 | G/A/T | R/S | 166 |
| rs772251328 | X:15425989 | G/T | S/Y | 161 |
| rs749315170 | X:15455852 | A/G | I/T | 159 |
| rs770779063 | X:15455853 | T/C | I/V | 159 |
| rs1357879054 | X:15455874 | T/C | I/V | 152 |
| rs1294033379 | X:15455876 | A/T | V/D | 151 |
| rs1426035240 | X:15455877 | C/T | V/I | 151 |
| rs1165626236 | X:15455883 | C/T | V/I | 149 |
| rs776734253 | X:15455885 | G/A | T/I | 148 |
| rs1455900711 | X:15455891 | C/G | G/A | 146 |
| rs768819532 | X:15455894 | T/C | D/G | 145 |
| rs777036326 | X:15455895 | C/T | D/N | 145 |
| rs762108669 | X:15455903 | G/A/C | P/L | 142 |
| rs762108669 | X:15455903 | G/A/C | P/R | 142 |
| rs1376925618 | X:15455904 | G/A | P/S | 142 |
| rs765758233 | X:15455906 | T/G | K/T | 141 |
| rs773449841 | X:15455914 | T/A | E/D | 138 |
| rs908993255 | X:15455922 | T/A/G | S/C | 136 |
| rs908993255 | X:15455922 | T/A/G | S/R | 136 |
| rs1349318268 | X:15455930 | T/C | E/G | 133 |
| rs1996173 | X:15455942 | G/A | P/L | 129 |
| rs1329364366 | X:15455951 | A/G | M/T | 126 |
| rs1484554733 | X:15455981 | A/G | L/P | 116 |
| rs752062795 | X:15455985 | G/T | Q/K | 115 |
| rs1474215131 | X:15455999 | G/A | P/L | 110 |
| rs147114611 | X:15456000 | G/T | P/T | 110 |
| rs35715407 | X:15456011 | C/A/G/T | C/F | 106 |
| rs35715407 | X:15456011 | C/A/G/T | C/S | 106 |
| rs35715407 | X:15456011 | C/A/G/T | C/Y | 106 |
| rs777341679 | X:15456018 | T/A | M/L | 104 |
| rs562829699 | X:15456024 | C/T | A/T | 102 |
| rs1467570812 | X:15456027 | G/A | H/Y | 101 |
| rs757030530 | X:15456033 | T/C | I/V | 99 |
| rs778749014 | X:15456035 | C/T | G/D | 98 |
| rs140109164 | X:15456036 | C/T | G/S | 98 |
| rs201144539 | X:15456038 | C/T | R/Q | 97 |
| rs138504766 | X:15456039 | G/A | R/W | 97 |
| rs149497039 | X:15456044 | G/A | A/V | 95 |
| rs866898423 | X:15459660 | C/G/T | L/F | 90 |
| rs763677923 | X:15459675 | C/T | M/I | 85 |
| rs1464579620 | X:15459682 | C/T | G/D | 83 |
| rs757045955 | X:15459688 | T/C/G | H/R | 81 |
| rs757045955 | X:15459688 | T/C/G | H/P | 81 |
| rs1042818236 | X:15459698 | A/C | F/V | 78 |
| rs750390136 | X:15459699 | G/T | D/E | 77 |
| rs1453112002 | X:15459713 | T/C | M/V | 73 |
| rs1248681779 | X:15459719 | C/T | G/S | 71 |
| rs780078643 | X:15459721 | C/A/G | G/V | 70 |
| rs780078643 | X:15459721 | C/A/G | G/A | 70 |
| rs748257098 | X:15459722 | C/T | G/R | 70 |
| rs1001224772 | X:15459740 | C/T | V/I | 64 |
| rs1459166472 | X:15479739 | C/A | G/V | 60 |
| rs1319331957 | X:15479740 | C/T | G/S | 60 |
| rs188288097 | X:15479742 | C/G/T | R/P | 59 |
| rs188288097 | X:15479742 | C/G/T | R/Q | 59 |
| rs780168534 | X:15479745 | T/C | H/R | 58 |
| rs1356176104 | X:15479750 | A/C | H/Q | 56 |
| rs866843206 | X:15479755 | C/T | D/N | 55 |
| rs751509679 | X:15479770 | G/A | P/S | 50 |
| rs777893526 | X:15479776 | C/G | G/R | 48 |
| rs749537580 | X:15479781 | T/C | K/R | 46 |
| rs1277921319 | X:15479791 | C/G | D/H | 43 |
| rs1444445052 | X:15479792 | A/T | F/L | 42 |
| rs1355385636 | X:15479793 | A/T | F/Y | 42 |
| rs746253345 | X:15479805 | G/A | P/L | 38 |
| rs545530472 | X:15479812 | G/T | L/M | 36 |
| rs766252248 | X:15491175 | A/G | I/T | 28 |
| rs751602550 | X:15491176 | T/C | I/V | 28 |
| rs754937245 | X:15491184 | C/T | R/Q | 25 |
| rs1485928589 | X:15491185 | G/A | R/W | 25 |
| rs758349788 | X:15491188 | C/A | V/F | 24 |
| rs34149789 | X:15491190 | C/G | R/T | 23 |
| rs202040906 | X:15491193 | G/A | A/V | 22 |
| rs1477252543 | X:15491200 | C/G | V/L | 20 |
| rs372506134 | X:15491202 | C/G | G/A | 19 |
| rs1429642125 | X:15491205 | T/C | E/G | 18 |
| rs769214822 | X:15491208 | G/A/C | S/L | 17 |
| rs769214822 | X:15491208 | G/A/C | S/W | 17 |
| rs1392250707 | X:15491211 | T/G | Q/P | 16 |
| rs1323145592 | X:15491212 | G/T | Q/K | 16 |
| rs747719985 | X:15491217 | C/T | R/Q | 14 |
| rs202231007 | X:15491218 | G/A | R/W | 14 |
| rs773044136 | X:15491223 | A/T | L/H | 12 |
| rs762678425 | X:15491229 | G/A | S/L | 10 |
| rs201327459 | X:15491233 | G/C | L/V | 9 |
| rs1340870817 | X:15491240 | T/A | K/N | 6 |
| rs1195816578 | X:15491245 | T/G | K/Q | 5 |
