# Supplementary material for: A comprehensive in silico exploration of the impacts of missense variants on two different conformations of human pirin protein
Source: Bull Natl Res Cent. 2022 Jul 30;46(1):225. doi: 10.1186/s42269-022-00917-7 (PMC9362109; doi:10.1186/s42269-022-00917-7)
Supplement: Supplementary file 2 — Additional file 2: Table S2. Buried hydrogen bonds broken by pirin missense variants. [file 42269_2022_917_MOESM2_ESM.docx]

**Supplementary Table 2:** Buried hydrogen bonds broken by pirin missense variants

| **Variant** | **Donor^a^** | **Acceptor^a^** | **Distance (Å)** | **Type^b^** |
| --- | --- | --- | --- | --- |
| D43H | A0026-ARG NH2 | A0043-ASP OD2 | 2.7 | SS |
| R59P | A0059-ARG NH1 | A0270-ASP OD2 | 2.82 | SS |
|  | A0059-ARG NH2 | A0097-ARG O | 2.81 | SM |
|  | A0059-ARG NH2 | A0270-ASP OD2 | 3.83 | SS |
|  | A0059-ARG NH2 | A0276-ASN O | 3.09 | SM |
| G60V | A0125-LYS NZ | A0060-GLY O | 2.99 | SM |
| F78V | A0280-ARG NH2 | A0078-PHE O | 3.73 | SM |
| H101Y | A0056-HIS NE2 | A0101-HIS NE2 | 2.86 | SS |
|  | A0101-HIS NE2 | A0056-HIS NE2 | 2.86 | SS |
|  | A0058-HIS NE2 | A0101-HIS NE2 | 3.27 | SS |
|  | A0101-HIS NE2 | A0103-GLU OE2 | 3.28 | SS |
| D173G | A0066-TYR OH | A0173-ASP OD2 | 3.8 | SS |
|  | A0237-HIS NE2 | A0173-ASP OD1 | 2.5 | SS |
|  | A0175-LYS NZ | A0173-ASP OD2 | 2.67 | SS |

^a^Donor and Acceptor amino acid residues described using A denoting A chain, following number denotes amino acid position in the protein, following O, OD1, OD2, OE2, OH, NE2, NH1, NH2, NZ denotes functional group of the participating residues.

^b^Type of the Hydrogen bond is described with SS and SM. SS denotes Side-chain and Side-chain Hydrogen bond, SM denotes Side-chain and Main-chain Hydrogen bond
