# Supplementary material for: A comprehensive in silico exploration of the impacts of missense variants on two different conformations of human pirin protein
Source: Bull Natl Res Cent. 2022 Jul 30;46(1):225. doi: 10.1186/s42269-022-00917-7 (PMC9362109; doi:10.1186/s42269-022-00917-7)
Supplement: Supplementary file 3 — Additional file 3: Table S3. Effects of missense variants on metal binding of pirin according to MIB tool. [file 42269_2022_917_MOESM3_ESM.docx]

**Supplementary Table 3:** Effects of missense variants on metal binding of pirin according to MIB tool

| **Fe2+ Conformation** | | | | | | |  | **Fe3+ Conformation** | | | | | | |
| --- | --- | --- | --- | --- | --- | --- | --- | --- | --- | --- | --- | --- | --- | --- |
| **Variants^a^** | **Predicted best binding sites** | **Overall score** | **Scores at**  **metal binding residue positions** | | | |  | **Variants^a^** | **Predicted best binding sites** | **Overall score** | **Scores at**  **Metal binding residue positions** | | | |
|  |  |  | **56** | **58** | **101** | **103** |  |  |  |  | **56** | **58** | **101** | **103** |
| Wild-type | 56H , 58H , 101H , 103E | 3.49 | 6.83 | 6.83 | 6.834 | 6.834 |  | Wild type | 56H ,  101H ,  103E | 1.785 | 6.108 | 3.984 | 6.108 | 6.108 |
| G19A | 56H , 58H , 101H , 103E | 3.49 | 6.849 | 6.849 | 6.849 | 6.849 |  | G19A | 56H ,  101H ,  103E | 1.652 | 5.442 | 3.996 | 5.442 | 5.442 |
| V24F | 56H , 58H , 101H , 103E | 3.49 | 6.841 | 6.841 | 6.841 | 6.841 |  | V24F | 56H ,  101H ,  103E | 1.652 | 5.442 | 3.996 | 5.442 | 5.442 |
| R25W | 56H , 58H , 101H , 103E | 3.49 | 6.873 | 6.873 | 6.873 | 6.873 |  | R25W | 56H ,  101H ,  103E | 1.785 | 6.366 | 4.161 | 6.366 | 6.366 |
| I28T | 56H , 58H , 101H , 103E | 3.49 | 6.841 | 6.841 | 6.841 | 6.841 |  | I28T | 56H ,  101H ,  103E | 1.652 | 5.442 | 3.996 | 5.442 | 5.442 |
| P38L | 56H , 58H , 101H , 103E | 3.49 | 6.841 | 6.841 | 6.841 | 6.841 |  | P38L | 56H ,  101H ,  103E | 1.652 | 5.442 | 3.996 | 5.442 | 5.442 |
| D43H | 56H , 58H , 101H , 103E | 3.49 | 6.758 | 6.758 | 6.758 | 6.758 |  | D43H | 56H ,  101H ,  103E | 1.652 | 5.207 | 3.814 | 5.207 | 5.207 |
| **H56Q** | 56Q , 58H , 101H , 103E | 2.257 | 6.009 | 6.009 | 6.009 | 6.009 |  | **H56Q** | 75H ,  81H ,  101H | 1.047 | 3.576 | 3.722 | 4.277 | 3.576 |
| **H58R** | 56H , 58R , 101H , 103E | 2.527 | 6.07 | 6.07 | 6.07 | 6.07 |  | H58R | 56H ,  101H ,  103E | 1.652 | 5.674 | 3.211 | 5.674 | 5.674 |
| R59P | 56H , 58H , 101H , 103E | 3.49 | 6.841 | 6.841 | 6.841 | 6.841 |  | R59P | 56H ,  101H ,  103E | 1.652 | 5.442 | 3.996 | 5.442 | 5.442 |
| R59Q | 56H , 58H , 101H , 103E | 3.49 | 6.841 | 6.841 | 6.841 | 6.841 |  | R59Q | 56H ,  101H ,  103E | 1.652 | 5.442 | 3.996 | 5.442 | 5.442 |
| G60S | 56H , 58H , 101H , 103E | 3.49 | 6.841 | 6.841 | 6.841 | 6.841 |  | G60S | 56H ,  101H ,  103E | 1.652 | 5.442 | 3.996 | 5.442 | 5.442 |
| G60V | 56H , 58H , 101H , 103E | 3.49 | 6.841 | 6.841 | 6.841 | 6.841 |  | G60V | 56H ,  101H ,  103E | 1.652 | 5.442 | 3.996 | 5.442 | 5.442 |
| G70A | 56H , 58H , 101H , 103E | 3.49 | 6.841 | 6.841 | 6.841 | 6.841 |  | G70A | 56H ,  101H ,  103E | 1.652 | 5.442 | 3.996 | 5.442 | 5.442 |
| G70R | 56H , 58H , 101H , 103E | 3.49 | 6.841 | 6.841 | 6.841 | 6.841 |  | G70R | 56H ,  101H ,  103E | 1.652 | 5.442 | 3.996 | 5.442 | 5.442 |
| G70V | 56H , 58H , 101H , 103E | 3.49 | 6.841 | 6.841 | 6.841 | 6.841 |  | G70V | 56H ,  101H ,  103E | 1.652 | 5.442 | 3.996 | 5.442 | 5.442 |
| D77E | 56H , 58H , 101H , 103E | 3.49 | 6.903 | 6.903 | 6.903 | 6.903 |  | D77E | 56H ,  101H ,  103E | 1.652 | 5.442 | 3.996 | 5.442 | 5.442 |
| F78V | 56H , 58H , 101H , 103E | 3.49 | 6.841 | 6.841 | 6.841 | 6.841 |  | F78V | 56H ,  101H ,  103E | 1.652 | 5.435 | 3.991 | 5.435 | 5.435 |
| H81P | 56H , 58H , 101H , 103E | 3.49 | 7.039 | 7.039 | 7.039 | 7.039 |  | H81P | 56H ,  101H ,  103E | 1.652 | 5.655 | 4.159 | 5.655 | 5.655 |
| G83D | 56H , 58H , 101H , 103E | 3.49 | 6.784 | 6.784 | 6.784 | 6.784 |  | G83D | 56H ,  101H ,  103E | 1.652 | 5.432 | 3.987 | 5.432 | 5.432 |
| L90F | 56H , 58H , 101H , 103E | 3.49 | 6.841 | 6.841 | 6.841 | 6.841 |  | L90F | 56H ,  101H ,  103E | 1.652 | 5.442 | 3.996 | 5.442 | 5.442 |
| A95V | 56H , 58H , 101H , 103E | 3.49 | 6.842 | 6.842 | 6.842 | 6.842 |  | A95V | 56H ,  101H ,  103E | 1.652 | 5.442 | 3.996 | 5.442 | 5.442 |
| G98D | 56H , 58H , 101H , 103E | 3.49 | 6.805 | 6.805 | 6.805 | 6.805 |  | G98D | 56H ,  101H ,  103E | 1.652 | 5.435 | 3.991 | 5.435 | 5.435 |
| G98S | 56H , 58H , 101H , 103E | 3.49 | 6.832 | 6.832 | 6.832 | 6.832 |  | G98S | 56H ,  101H ,  103E | 1.652 | 5.435 | 3.991 | 5.435 | 5.435 |
| **H101Y** | 56H , 58H , 101Y , 103E | 2.768 | 6.49 | 6.49 | 6.49 | 6.49 |  | **H101Y** | 56H ,  101Y ,  103E | 1.162 | 4.38 | 4.277 | 4.38 | 4.38 |
| Q115K | 56H , 58H , 101Y , 103E | 3.49 | 6.853 | 6.853 | 6.853 | 6.853 |  | Q115K | 56H ,  101H ,  103E | 1.652 | 5.375 | 3.944 | 5.375 | 5.375 |
| L116P | 56H , 58H , 101Y , 103E | 3.49 | 6.85 | 6.85 | 6.85 | 6.85 |  | L116P | 56H ,  101H ,  103E | 1.652 | 5.442 | 3.996 | 5.442 | 5.442 |
| M126T | 56H , 58H , 101Y , 103E | 3.49 | 6.841 | 6.841 | 6.841 | 6.841 |  | M126T | 56H ,  101H ,  103E | 1.652 | 5.442 | 3.996 | 5.442 | 5.442 |
| P129L | 56H , 58H , 101Y , 103E | 3.49 | 6.841 | 6.841 | 6.841 | 6.841 |  | P129L | 56H ,  101H ,  103E | 1.652 | 5.442 | 3.996 | 5.442 | 5.442 |
| V151D | 56H , 58H , 101Y , 103E | 3.49 | 6.841 | 6.841 | 6.841 | 6.841 |  | V151D | 56H ,  101H ,  103E | 1.652 | 5.442 | 3.996 | 5.442 | 5.442 |
| S161Y | 56H , 58H , 101Y , 103E | 3.49 | 6.859 | 6.859 | 6.859 | 6.859 |  | S161Y | 56H ,  101H ,  103E | 1.785 | 6.366 | 4.161 | 6.366 | 6.366 |
| T167I | 56H , 58H , 101Y , 103E | 3.49 | 6.841 | 6.841 | 6.841 | 6.841 |  | T167I | 56H ,  101H ,  103E | 1.652 | 5.442 | 3.996 | 5.442 | 5.442 |
| D173G | 56H , 58H , 101Y , 103E | 3.49 | 6.856 | 6.856 | 6.856 | 6.856 |  | D173G | 56H ,  101H ,  103E | 1.652 | 5.468 | 4.017 | 5.468 | 5.468 |
| D173N | 56H , 58H , 101Y , 103E | 3.49 | 6.841 | 6.841 | 6.841 | 6.841 |  | D173N | 56H ,  101H ,  103E | 1.652 | 5.468 | 4.017 | 5.468 | 5.468 |
| G179V | 56H , 58H , 101Y , 103E | 3.49 | 6.841 | 6.841 | 6.841 | 6.841 |  | G179V | 56H ,  101H ,  103E | 1.652 | 5.237 | 3.837 | 5.237 | 5.237 |
| P187L | 56H , 58H , 101Y , 103E | 3.49 | 6.841 | 6.841 | 6.841 | 6.841 |  | P187L | 56H ,  101H ,  103E | 1.652 | 5.442 | 3.996 | 5.442 | 5.442 |
| W190S | 56H , 58H , 101Y , 103E | 3.49 | 6.841 | 6.841 | 6.841 | 6.841 |  | W190S | 56H ,  101H ,  103E | 1.652 | 5.442 | 3.996 | 5.442 | 5.442 |
| L220P | 56H , 58H , 101Y , 103E | 3.49 | 6.841 | 6.841 | 6.841 | 6.841 |  | L220P | 56H ,  101H ,  103E | 1.652 | 5.442 | 3.996 | 5.442 | 5.442 |
| P245S | 56H , 58H , 101Y , 103E | 3.49 | 6.841 | 6.841 | 6.841 | 6.841 |  | P245S | 56H ,  101H ,  103E | 1.652 | 5.442 | 3.996 | 5.442 | 5.442 |
| E248A | 56H , 58H , 101Y , 103E | 3.49 | 6.841 | 6.841 | 6.841 | 6.841 |  | E248A | 56H ,  101H ,  103E | 1.652 | 5.654 | 4.16 | 5.654 | 5.654 |
| E248D | 56H , 58H , 101Y , 103E | 3.49 | 6.841 | 6.841 | 6.841 | 6.841 |  | E248D | 56H ,  101H ,  103E | 1.652 | 5.58 | 4.102 | 5.58 | 5.58 |
| G254C | 56H , 58H , 101Y , 103E | 3.49 | 6.868 | 6.868 | 6.868 | 6.868 |  | G254C | 56H ,  101H ,  103E | 1.652 | 5.396 | 3.96 | 5.396 | 5.396 |
| G254V | 56H , 58H , 101Y , 103E | 3.49 | 6.85 | 6.85 | 6.85 | 6.85 |  | G254V | 56H ,  101H ,  103E | 1.652 | 5.442 | 3.996 | 5.442 | 5.442 |
| V257A | 56H , 58H , 101Y , 103E | 3.49 | 6.841 | 6.841 | 6.841 | 6.841 |  | V257A | 56H ,  101H ,  103E | 1.652 | 5.442 | 3.996 | 5.442 | 5.442 |
| M258I | 56H , 58H , 101Y , 103E | 3.49 | 6.841 | 6.841 | 6.841 | 6.841 |  | M258I | 56H ,  101H ,  103E | 1.652 | 5.442 | 3.996 | 5.442 | 5.442 |
| I264S | 56H , 58H , 101Y , 103E | 3.49 | 6.841 | 6.841 | 6.841 | 6.841 |  | I264S | 56H ,  101H ,  103E | 1.785 | 6.108 | 3.984 | 6.108 | 6.108 |

^a^Variants that exhibited noticeable changes in binding score compared to wild-type are written in bold letters
